# The knockdown of *OsVIT2* and *MIT* affects iron localization in rice seed

**DOI:** 10.1186/1939-8433-6-31

**Published:** 2013-11-20

**Authors:** Khurram Bashir, Ryuichi Takahashi, Shamim Akhtar, Yasuhiro Ishimaru, Hiromi Nakanishi, Naoko K Nishizawa

**Affiliations:** Graduate School of Agricultural and Life Sciences, The University of Tokyo, 1-1-1 Yayoi, Bunkyo-ku, Tokyo, 113-8657 Japan; Faculty of Science, Graduate School of Science, Tohoku University, 6-3 Aramakiaza-aoba, Aoba-ku, Sendai, Miyagi, 980-8578 Japan; Research Institute for Bioresources and Biotechnology, Ishikawa Prefectural University, 1-308 Suematsu, Nonoichi-shi, Ishikawa, 921-8836 Japan; Plant Genomic Network Research Team, Center for Sustainable Resource Sciences, RIKEN Yokohama Campus, 1-7-22, Suehiro-cho, Tsurumi-ku, Yokohama, Kanagawa, 230-0045 Japan

**Keywords:** Iron, Manganese, Mitochondrial iron transporter, *Oryza sativa*, Vacuolar iron transporter, Zinc

## Abstract

**Background:**

The mechanism of iron (Fe) uptake in plants has been extensively characterized, but little is known about how Fe transport to different subcellular compartments affects Fe localization in rice seed. Here, we discuss the characterization of a rice vacuolar Fe transporter 2 (*OsVIT2*) T-DNA insertion line (*osvit2*) and report that the knockdown of *OsVIT2* and mitochondrial Fe transporter (*MIT*) expression affects seed Fe localization.

**Findings:**

*osvit2* plants accumulated less Fe in their shoots when grown under normal or excess Fe conditions, while the accumulation of Fe was comparable to that in wild-type (WT) plants under Fe-deficient conditions. The accumulation of zinc, copper, and manganese also changed significantly in the shoots of *osvit2* plants. The growth of *osvit2* plants was also slow compared to that of WT plants. The concentration of Fe increased in *osvit2* polished seeds. Previously, we reported that the expression of *OsVIT2* was higher in *MIT* knockdown (*mit-2*) plants, and in this study, the accumulation of Fe in *mit-2* seeds decreased significantly.

**Conclusions:**

These results suggest that vacuolar Fe trafficking is important for plant Fe homeostasis and distribution, especially in plants grown in the presence of excess Fe. Moreover, changes in the expression of *OsVIT2* and *MIT* affect the concentration and localization of metals in brown rice as well as in polished rice seeds.

**Electronic supplementary material:**

The online version of this article (doi:10.1186/1939-8433-6-31) contains supplementary material, which is available to authorized users.

## Findings

Iron (Fe) is an essential micronutrient for all higher organisms. Plants require Fe for several cellular processes, including respiration, chlorophyll biosynthesis, and photosynthetic electron transport (Marschner 1995). The molecular mechanism of Fe transport in rice has been well documented (Bashir et al. 2006; Bashir and Nishizawa 2006; Bashir et al. 2010; Bashir et al. 2011b; Bashir et al. 2013a; Ishimaru et al. 2012; Kobayashi and Nishizawa 2012). Once inside a plant, Fe enters root cells and is transported to the shoot and seeds. Fe performs vital roles in subcellular organelles such as chloroplasts and mitochondria, and defects in mitochondrial Fe homeostasis significantly affect plant growth (Bashir et al. 2011a; Bashir et al. 2011c; Ishimaru et al. 2009). As excess Fe in the cytoplasm may be toxic, it is either stored as ferritin in chloroplasts or is diverted to the vacuole. Knockout mutants for the rice vacuolar metal transporters *OsVIT1* and *OsVIT2* were recently reported to accumulate increased amounts of Fe in their seeds (Zhang et al. 2012). This accumulation was mainly observed in the embryo (Zhang et al. 2012), which is removed during milling. In this short report, we describe the characterization of a mutant in which the expression of *OsVIT2* was knocked down and we show that changes in the expression of *OsVIT2* and mitochondrial iron transporter (*MIT*) affect seed Fe localization in brown rice as well as in polished rice seeds.

**Figure 1:**
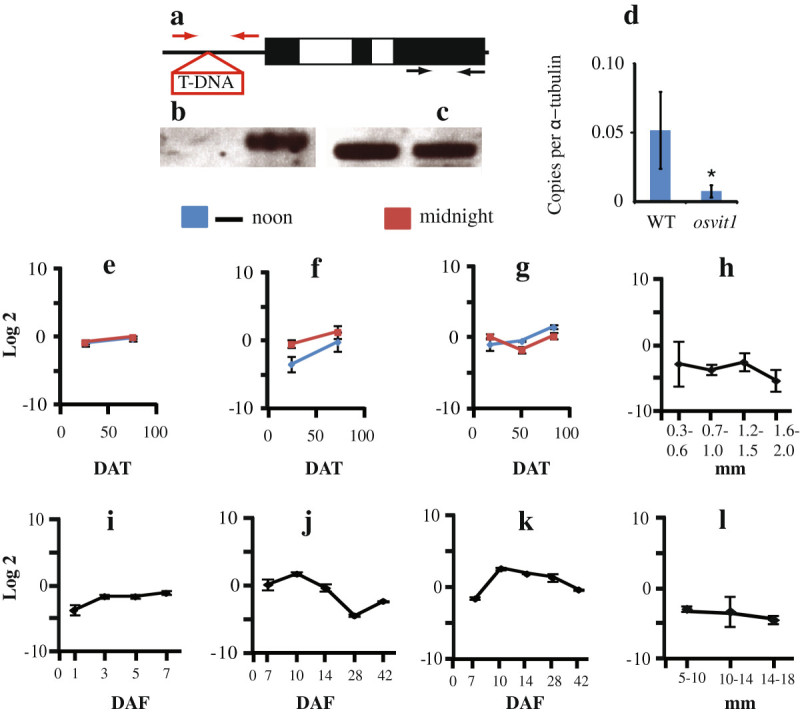
**Characterization of the** ***OsVIT2*** **knockdown mutant (** ***Osvit2*** **) and expression of** ***OsVIT2*** **during different developmental stages of rice. a**: Gene structure and positions of the primers specific for the T-DNA insertion. **b**: Bands corresponding to the primers are shown in red. **c**: Bands corresponding to the primers are shown black. **d**: Quantitative reverse-transcription PCR analysis showing the expression of *Osvit2* in WT and knockdown plants. A vertical bar followed by an asterisk indicates a significant difference from WT according to the Tukey–Kramer test (p < 0.05; WT, n = 4; *osvit2*, n = 8). **e**: Roots at 27 and 76 DAT. **f**: Stems at 27 and 76 DAT. **g**: Leaf blades at 27, 76, and 125 DAT. **h**: The anther size was 0.3–0.6, 0.7–1.0, 1.2–1.5, and 1.6–2.0 mm. **i**: Embryos 1, 3, 5, and 7 days after flowering. **j**: Ovaries 7, 10, 14, 28, and 42 days after flowering. **k**: Endosperm, 7, 10, 14, 28, and 42 days after flowering. **l**: The pistil size was 5–10, 10–14, and 14–18 mm (n = 3). Error bars represent the SD.

We characterized a T-DNA line (An et al. 2003a; Jeong et al. 2006) in which the T-DNA was integrated ~500 bp upstream of the start codon of *OsVIT2* (Os09g0396900), as shown in Figure 1a. (For details see Additional file 1 “Methods”). Genomic polymerase chain reaction (PCR) using primers specific for the T-DNA integration site confirmed the homozygous status of the plants (Figure 1b), while primers specific for exon 3 were used to check the quality of the DNA (Figure 1c). Quantitative PCR analysis confirmed that the expression of *OsVIT2* was significantly downregulated in the *osvit2* line compared to wild-type (WT) plants (Figure 1d). Note that similar to indica rice, the sequence of *OsVIT2* is not complete in RAP-DB (http://rapdb.dna.affrc.go.jp/) and the sequence of *OsVIT2* showed 100% similarity to that reported for indica rice (Zhang et al. 2012). Data related to the expression of *OsVIT2* were generated through rice global gene expression profile data sets maintained at http://ricexpro.dna.affrc.go.jp (Sato et al. 2011a; Sato et al. 2011b). *OsVIT2* expression is upregulated in the presence of excess Fe (Bashir et al. 2011c), and the expression of *OsVIT2* could be observed through all developmental stages (Figure 1e–l). In roots, the expression of *OsVIT* 2 increased slightly from 27 days after transplantation (DAT) to 76 DAT and was not regulated diurnally (Figure 1e). A similar trend was observed in the stems (Figure 1f). In leaves, the expression of *OsVIT2* increased slightly at noon from 27 to 76 DAT and at 125 DAT, while at midnight, it decreased slightly from 27 to 76 DAT and then increased at 127 DAT (Figure 1g). *OsVIT2* expression did not change significantly during anther development (Figure 1h). In the embryo, *OsVIT2* expression increased slightly from 1 to 3, 5, and 7 days after fertilization (DAF; Figure 1i). In ovary, *OsVIT2* expression first increased from 7 to 10 DAF, then decreased from 10 to 14 and 28 DAF, and then increased again at 42 DAF (Figure 1j). In endosperm, the expression of *OsVIT2* first increased from 7 to 10 DAF and then decreased from 10, 14, 28, and 42 DAF (Figure 1k). In addition, *OsVIT2* expression remained largely unchanged during pistil development (Figure 1l). These results clearly support the earlier results of Zhang et al. (2012) showing that *OsVIT2* plays a critical role in transporting Fe and zinc (Zn) from leaves to seeds.

**Figure 2:**
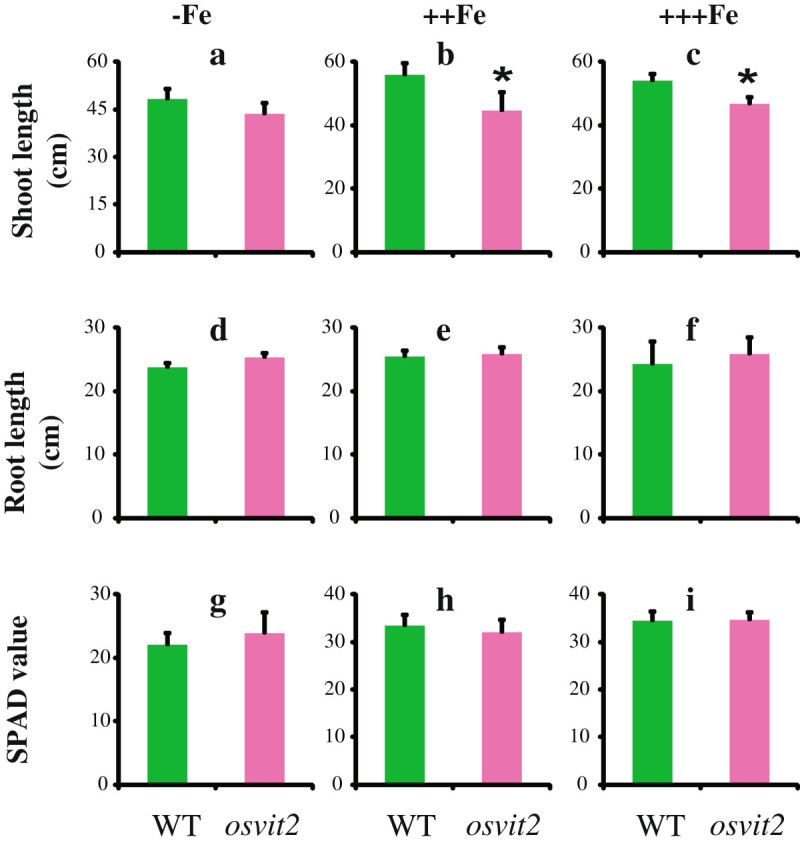
**Morphological characteristics of** ***osvit2*** **plants grown at different Fe concentrations. a–c**: Shoot length. **d–f**: Root length. **g–i**: SPAD value. **a, d,** and **g**: –Fe (0 μM Fe-EDTA). **b, e,** and **h**: +Fe (100 μM Fe-EDTA). **c, f,** and **i**: ++Fe (500 μM Fe-EDTA). Vertical bars followed by an asterisk indicate a significant difference from the WT according to the Tukey–Kramer test (p < 0.05; n = 4).

**Figure 3:**
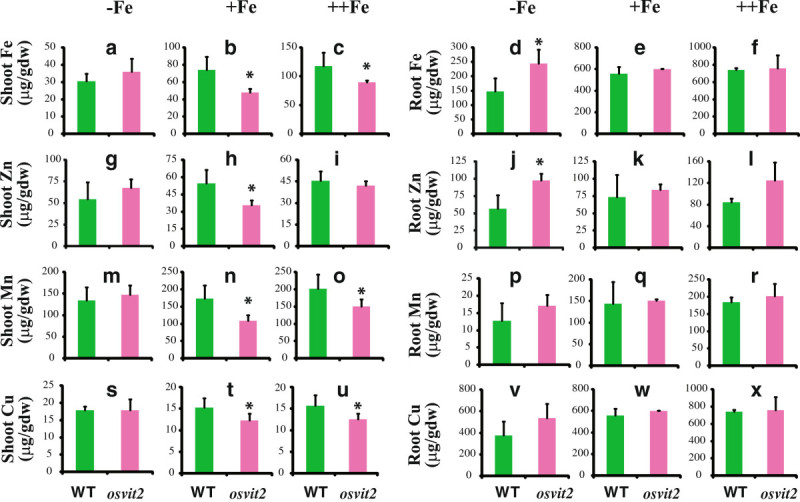
**Metal concentration of** ***osvit2*** **plants grown at different Fe concentrations. a–c**: Shoot Fe concentration. **d–f**: Fe concentration. **g–i**: Shoot Zn concentration. **j–l**: Root Zn concentration. **m–o**: Shoot Mn concentration. **p–r**: Root Mn concentration. **s–u**: Shoot Cu concentration. **v–x**: Root Cu concentration. **a, d, g, j, m, p, s,** and **v**: –Fe (0 μM Fe-EDTA). **b, e, h, k, n, q, t,** and **w**: +Fe (100 μM Fe-EDTA). **c, f, i, l, o, r, u,** and **x**: ++Fe (500 μM Fe-EDTA). Vertical bars followed by an asterisk indicate a significant difference from the WT according to the Tukey–Kramer test (p < 0.05; n = 4).

**Figure 4:**
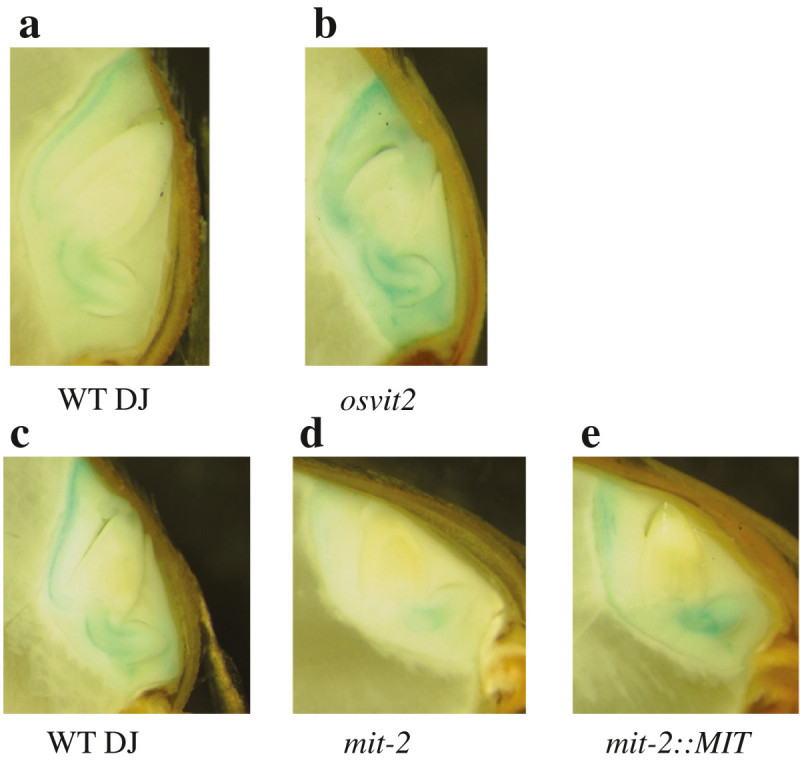
**Localization of Fe in WT,** ***osvit2*** **,** ***mit-2*** **, and** ***mit-2:MIT*** **lines. a**: WT DJ. **b**: *osvit2*. **c**: WT DJ. **d**: *mit-2*. **e**: *mit-2* complemented with the *MIT* open reading frame.

We grew WT and *osvit2* plants under different Fe concentrations. Under Fe-deficient conditions, no difference was observed in shoot length, root length, or chlorophyll content (SPAD value; Figure 2a, d, and g), while at 100 and 500 μΜ Fe-EDTA, shoot growth was significantly retarded compared to WT plants (Figure 2b and c), whereas the root length and SPAD value remained unchanged (Figure 2e, f, h, and i). We also measured the concentrations of Fe, Zn, manganese (Mn), and copper (Cu) in the roots and shoots of WT and *osvit2* plants. No difference was observed between WT and *osvit2* shoots for all of these metals when the plants were grown in the absence of Fe (Figure 3a, g, m, and s). When plants were grown in the presence of 100 μΜ Fe-EDTA, the concentrations of Fe, Zn, and Mn decreased significantly in *osvit2* compared to WT plants. The concentration of Cu also decreased. When plants were grown under Fe excess conditions, the concentrations of Fe, Mn, and Cu decreased significantly in the shoots of *osvit2* plants. The roots of *osvit2* plants accumulated more Fe and Zn when grown under Fe-deficient conditions (Figure 3d and j), while the metal concentration in *osvit2* roots was comparable to that in WT plants following growth with 100 or 500 μM Fe-EDTA (Figure 3e, f, k, l, p–r, and v–x). Pearl staining analysis showed that *osvit2* plants accumulated more Fe in their embryos compared to WT plants (Figure 4a and b). Mutants for *AtVIT1* (Kim et al. 2006) and *OsVIT1* and *OsVIT2* (Zhang et al. 2012) have been reported to have disturbed Fe accumulation in seeds.

**Figure 5:**
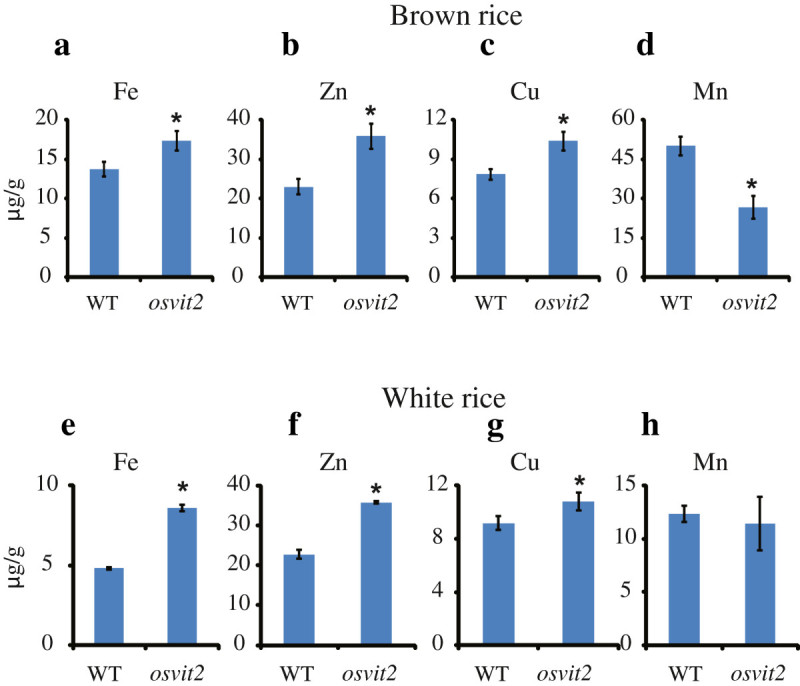
**Measurement of the metal concentration in WT and** ***osvit2*** **brown rice and polished seeds. a–d**: Brown rice. **e** and **f**: Polished rice. **a** and **e**: Fe concentration. **b** and **f**: Zn concentration. **c** and **g**: Cu concentration. **d** and **h**: Mn concentration. Vertical bars followed by an asterisk indicate a significant difference from the WT according to the Tukey–Kramer test (p < 0.05; n = 4).

**Figure 6:**
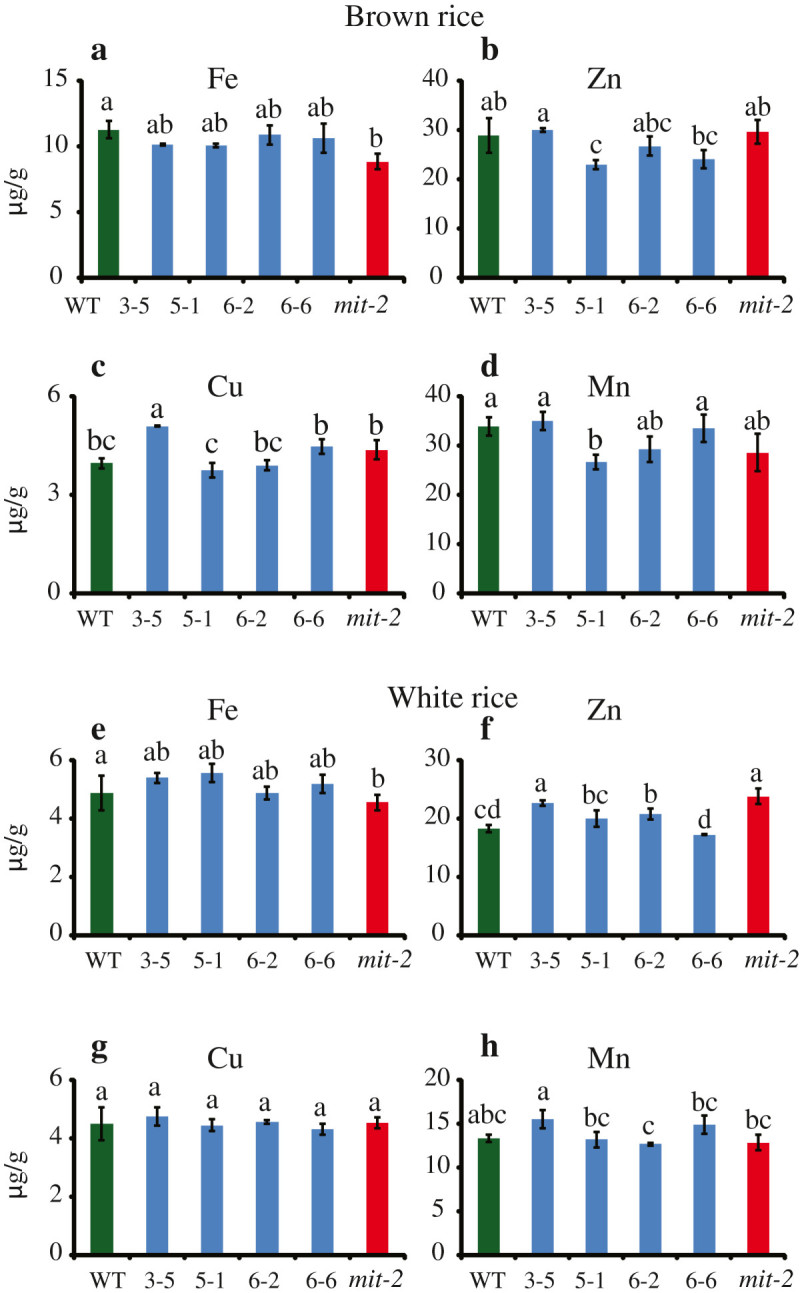
**Measurement of the metal concentration in WT and** ***mit-2*** **brown rice and polished seeds. a–d**: Brown rice. **e** and **f**: Polished rice. **a** and **e**: Fe concentration. **b** and **f**: Zn concentration. **c** and **g**: Cu concentration. **d** and **h**: Mn concentration. Vertical bars followed by different letters are significantly different from each other according to the Tukey–Kramer test (p < 0.05; n = 4).

We previously reported that in *mit-2* plants, the expression of *OsVIT2* was upregulated (Bashir et al. 2011c), so we assessed whether the concentration of Fe and other metals also changed in *mit-2* seeds. *mit-2* seeds accumulated less Fe in the embryo compared to WT plants, and in *mit-2* lines complemented with *MIT*, the localization of Fe was comparable to that in WT plants. We further measured the concentration of different metals in *mit-2* and *osvit2* seeds. The concentrations of Fe, Zn, and Cu increased in *osvit2* seeds, while that of Mn decreased (Figure 5). As we previously reported that the expression of *OsVIT2* was upregulated in *mit-2* plants (Bashir et al. 2011c), we analyzed *mit-2* seeds for changes in metal accumulation. The concentration of Fe was higher in leaves harvested from *mit-2* plants. A knockout line of *osvit2* was previously shown to accumulate an increased amount of Fe in its seeds (Zhang et al. 2012); however, in that report, the authors did not analyze the metal concentration in polished rice. As the embryo is removed during milling, leaving the endosperm as the only edible part, we measured the concentration of Fe and other metals in polished rice seeds (rice endosperm). In polished *osvit2* seeds, the concentrations of Fe, Zn, and Cu were significantly elevated compared to WT seeds (Figure 5e–g). Rice is an important agronomical crop and is used as a staple food by approximately half of the world’s population. Rice is poor in nutrients such as Fe, and people who depend on rice as a staple food often suffer from Fe deficiency (Bashir et al. 2013b; Bashir et al. 2010). Fe and Zn deficiencies cause 0.8 million deaths annually, while the number of people suffering from these deficiencies is up to 2 billion (World Health Organization 2003). Thus, breeding rice plants that are capable of accumulating more Fe and Zn in the endosperm is important (Bashir et al. 2012; Bashir et al. 2013b). These results indicate that the knockout/knockdown of *osvit2* may be utilized for biofortification programs. In *mit-2* seeds, Fe accumulation was significantly lower compared to WT plants (Figure 6a), while the concentration of other metals did not change significantly (Figure 6a–d). Polished *mit-2* seeds also accumulated significantly less Fe, while they accumulated more Zn compared to WT seeds (Figure 6e and f); no difference was observed in other metals. *OsVIT2* overexpression lines accumulated less Fe in their seeds, and in *mit-2*, the reduction in Fe accumulation may be caused by increased *OsVIT2* expression. Signaling between different subcellular organelles (Vigani et al. 2013) may be responsible for changes in metal localization in rice seeds. These results suggest that subcellular Fe transporters affect seed Fe localization; thus, it may be possible to regulate the expression of these transporters to biofortify rice with Fe and Zn without causing any adverse effects on plant growth and development.

## Electronic supplementary material

Additional file 1: Methods. (DOCX 19 KB)

Below are the links to the authors’ original submitted files for images.

Authors’ original file for figure 1

Authors’ original file for figure 2

Authors’ original file for figure 3

Authors’ original file for figure 4

Authors’ original file for figure 5

Authors’ original file for figure 6

## References

[CR1] An S, Park S, Jeong D-H, Lee D-Y, Kang H-G, Yu J-H, Hur J, Kim S-R, Kim Y-H, Lee M, Han S, Kim S-J, Yang J, Kim E, Wi SJ, Chung HS, Hong J-P, Choe V, Lee H-K, Choi J-H, Nam J, Kim S-R, Park P-B, Park KY, Kim WT, Choe S, Lee C-B, An G (2003a). Generation and analysis of end aequence satabase for T-DNA tagging lines in rice. Plant Physiol.

[CR2] Bashir K, Nishizawa NK (2006). Deoxymugineic acid synthase: a gene important for fe-acquisition and homeostasis. Plant Signal Behav.

[CR3] Bashir K, Inoue H, Nagasaka S, Takahashi M, Nakanishi H, Mori S, Nishizawa NK (2006). Cloning and characterization of deoxymugineic acid synthase genes from graminaceous plants. J Biol Chem.

[CR4] Bashir K, Ishimaru Y, Nishizawa NK (2010). Iron uptake and loading into rice grains. Rice.

[CR5] Bashir K, Ishimaru Y, Nishizawa NK (2011). Identification and characterization of the major mitochondrial Fe transporter in rice. Plant Signal Behav.

[CR6] Bashir K, Ishimaru Y, Shimo H, Kakei Y, Senoura T, Takahashi R, Sato Y, Sato Y, Uozumi N, Nakanishi H, Nishizawa NK (2011). Rice phenolics efflux transporter 2 (PEZ2) plays an important role in solubilizing apoplasmic iron. Soil Sci Plant Nutr.

[CR7] Bashir K, Ishimaru Y, Shimo H, Nagasaka S, Fujimoto M, Takanashi H, Tsutsumi N, An G, Nakanishi H, Nishizawa NK (2011). The rice mitochondrial iron transporter is essential for plant growth. Nat Commun.

[CR8] Bashir K, Ishimaru Y, Nishizawa NK (2012). Molecular mechanisms of zinc uptake and translocation in rice. Plant Soil.

[CR9] Bashir K, Nozoye T, Ishimaru Y, Nakanishi H, Nishizawa NK (2013a). Exploiting new tools for iron bio-fortification of rice: biotechnology advances.

[CR10] Bashir K, Takahashi R, Nakanishi H, Nishizawa NK (2013). The road to micronutrient biofortification of rice: progress and prospects. Front Plant Sci.

[CR11] Ishimaru Y, Bashir K, Fujimoto M, An G, Itai RN, Tsutsumi N, Nakanishi H, Nishizawa NK (2009). Rice-specific mitochondrial iron-regulated gene (MIR) plays an important role in iron homeostasis. Mol Plant.

[CR12] Ishimaru Y, Takahashi R, Bashir K, Shimo H, Senoura T, Sugimoto K, Ono K, Yano M, Ishikawa S, Arao T, Nakanishi H, Nishizawa NK (2012). Characterizing the role of rice NRAMP5 in manganese, iron and cadmium transport. Sci Rep.

[CR13] Jeong D-H, An S, Park S, Kang H-G, Park G-G, Kim S-R, Sim J, Kim Y-O, Kim M-K, Kim S-R, Kim J, Shin M, Jung M, An G (2006). Generation of a flanking sequence-tag database for activation-tagging lines in japonica rice. Plant J.

[CR14] Kim SA, Punshon T, Lanzirotti A, Li L, Alonso JM, Ecker JR, Kaplan J, Guerinot ML (2006). Localization of iron in Arabidopsis seed requires the vacuolar membrane transporter VIT1. Science.

[CR15] Kobayashi T, Nishizawa NK (2012). Iron uptake, translocation, and regulation in higher plants. Annu Rev Plant Biol.

[CR16] Marschner H (1995). Mineral nutrition of higher plants.

[CR17] Sato Y, Antonio B, Namiki N, Motoyama R, Sugimoto K, Takehisa H, Minami H, Kamatsuki K, Kusaba M, Hirochika H, Nagamura Y (2011). Field transcriptome revealed critical developmental and physiological transitions involved in the expression of growth potential in japonica rice. BMC Plant Biol.

[CR18] Sato Y, Antonio BA, Namiki N, Takehisa H, Minami H, Kamatsuki K, Sugimoto K, Shimizu Y, Hirochika H, Nagamura Y (2011). RiceXPro: a platform for monitoring gene expression in japonica rice grown under natural field conditions. Nucleic Acids Res.

[CR19] Vigani G, Zocchi G, Bashir K, Philippar K, Briat J-F (2013). Signals from chloroplasts and mitochondria for iron homeostasis regulation. Trends Plant Sci.

[CR20] World Health Organization (2003). Summary and conclusion of the sixty-first meeting of the joint FAO/WHO expert committee on food additives.

[CR21] Zhang Y, Xu Y-H, Yi H-Y, Gong J-M (2012). Vacuolar membrane transporters OsVIT1 and OsVIT2 modulate iron translocation between flag leaves and seeds in rice. Plant J.

