# Supplementary material for: The knockdown of OsVIT2 and MIT affects iron localization in rice seed
Source: Rice (N Y). 2013 Nov 20;6:31. doi: 10.1186/1939-8433-6-31 (PMC4883708; doi:10.1186/1939-8433-6-31)
Supplement: Supplementary file 1 — Additional file 1: Methods. (DOCX 19 KB) [file 12284_2013_65_MOESM1_ESM.docx]

**Methods**

The integration of T-DNA was confirmed with forward and revers primers as 5`- TTCAGAGCGGTTGAAACCCG-3`and 5`-CTGGACGAGCACACCGAGAA-3` respectively, while for the confirmation of DNA quality 5`-TAGGTATCTGGCGGCGAAGA-3`and 5`-GTGTATTAGATGTTCTGGAGGTG-3` were used as forward and reverse primers.

For *osvit2* real time RT PCR analysis 5`-AAGGCCTGGCTCGAATTCATG-3` and 5`-GTGTATTAGATGTTCTGGAGGTG-3` were used as forward and reverse primers respectively. The primers used for internal control in RT-PCR were α-tubulin forward, (5′-TCTTCCACCCTGAGCAGCTC-3′) and *α-tubulin* reverse (5′-AACCTTGGAGACCAGTGCAG-3′). The data regarding expression of *OsVIT2* was generated through rice global gene expression profile data sets ([Sato et al. 2011a](#_ENREF_2); [Sato et al. 2011b](#_ENREF_3)) (<http://www.ricexpro.dna.affrc.go.jp/index.html>). *mit-2* and *mit-2:MIT* complemented lines has already been reported (Bashir et al., 2011c). Pearl staining were done as described ([Zhang et al. 2012](#_ENREF_4)), and the metal concentration were measured as described previously ([Bashir et al. 2011](#_ENREF_1))

**References**

Bashir K, Ishimaru Y, Shimo H, Nagasaka S, Fujimoto M, Takanashi H, Tsutsumi N, An G, Nakanishi H, Nishizawa NK (2011) The rice mitochondrial iron transporter is essential for plant growth. Nature Communications 2:322. doi:10.1038/ncomms1326

Sato Y, Antonio B, Namiki N, Motoyama R, Sugimoto K, Takehisa H, Minami H, Kamatsuki K, Kusaba M, Hirochika H, Nagamura Y (2011a) Field transcriptome revealed critical developmental and physiological transitions involved in the expression of growth potential in japonica rice. BMC Plant Biology 11 (1):10

Sato Y, Antonio BA, Namiki N, Takehisa H, Minami H, Kamatsuki K, Sugimoto K, Shimizu Y, Hirochika H, Nagamura Y (2011b) RiceXPro: a platform for monitoring gene expression in japonica rice grown under natural field conditions. Nucleic Acids Research 39 (suppl 1):D1141-D1148. doi:10.1093/nar/gkq1085

Zhang Y, Xu Y-H, Yi H-Y, Gong J-M (2012) Vacuolar membrane transporters OsVIT1 and OsVIT2 modulate iron translocation between flag leaves and seeds in rice. The Plant Journal (72):400-410. doi:10.1111/j.1365-313X.2012.05088.x
