# Supplementary figures and images for: The knockdown of OsVIT2 and MIT affects iron localization in rice seed
Source: Rice (N Y). 2013 Nov 20;6:31. doi: 10.1186/1939-8433-6-31 (PMC4883708; doi:10.1186/1939-8433-6-31)

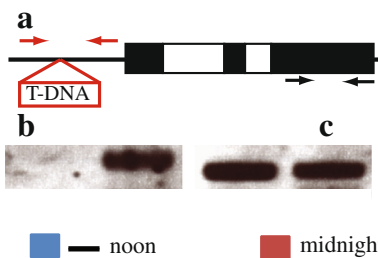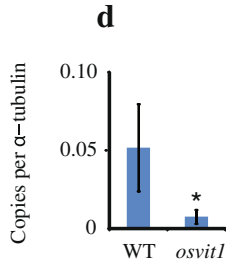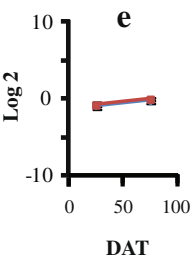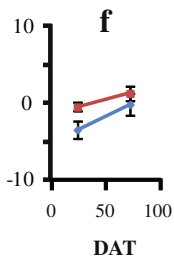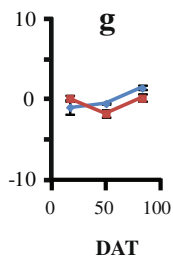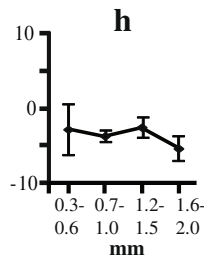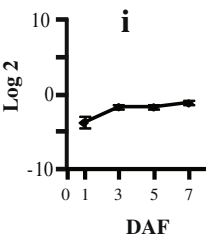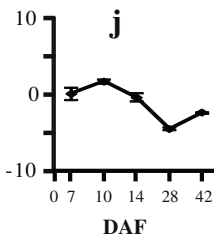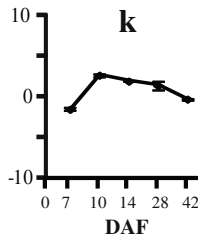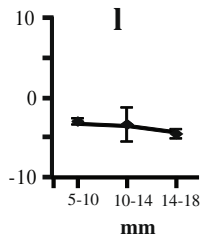

Supplement: Supplementary file 2 — Authors’ original file for figure 1 [file 12284_2013_65_MOESM2_ESM.pdf]

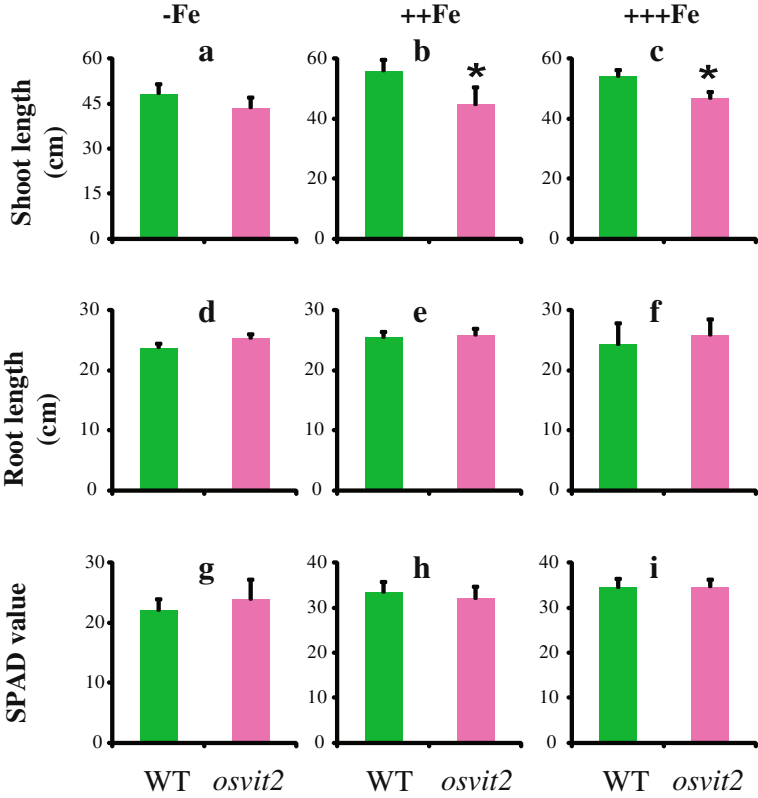

Supplement: Supplementary file 3 — Authors’ original file for figure 2 [file 12284_2013_65_MOESM3_ESM.pdf]

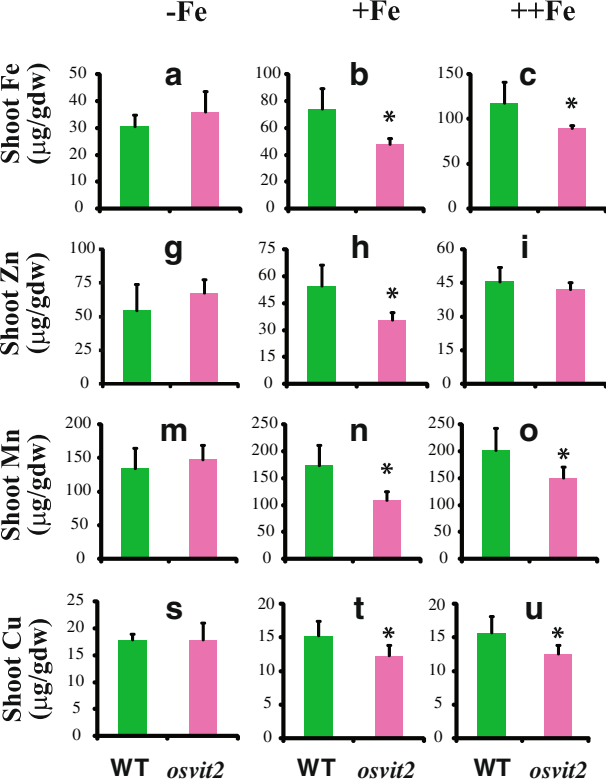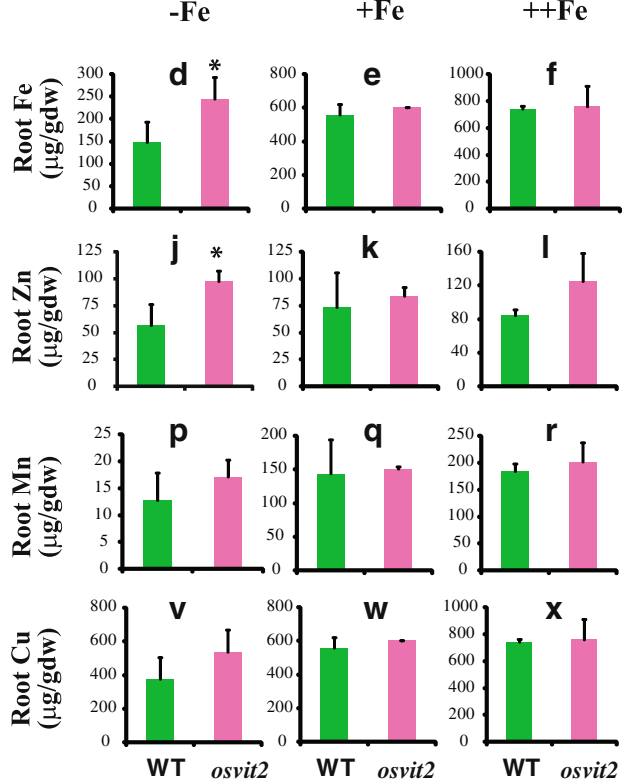

Supplement: Supplementary file 4 — Authors’ original file for figure 3 [file 12284_2013_65_MOESM4_ESM.pdf]

**a**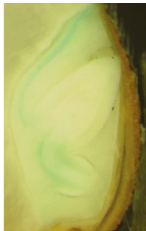

WT DJ

**b**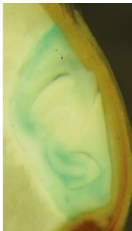*osvit2***c**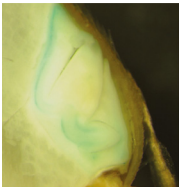

WT DJ

**d**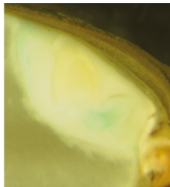*mit-2***e**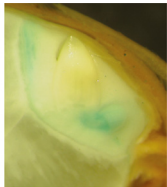*mit-2::MIT*

Supplement: Supplementary file 5 — Authors’ original file for figure 4 [file 12284_2013_65_MOESM5_ESM.pdf]

## Brown rice

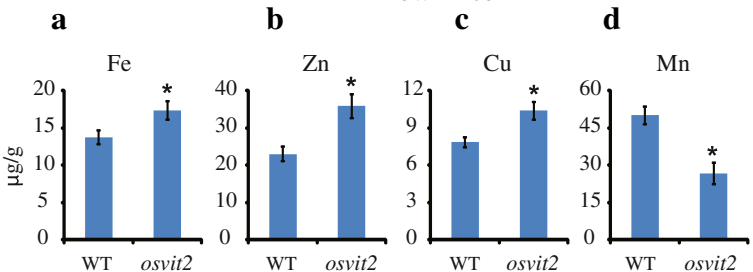

## White rice

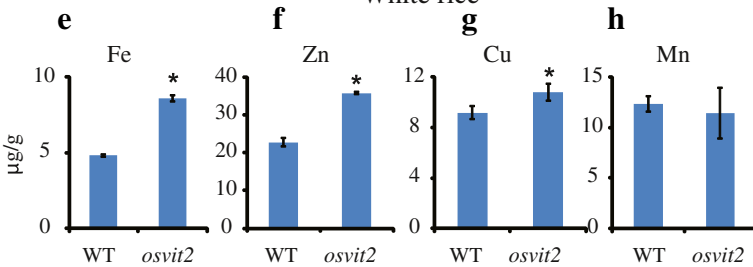

Supplement: Supplementary file 6 — Authors’ original file for figure 5 [file 12284_2013_65_MOESM6_ESM.pdf]

## Brown rice

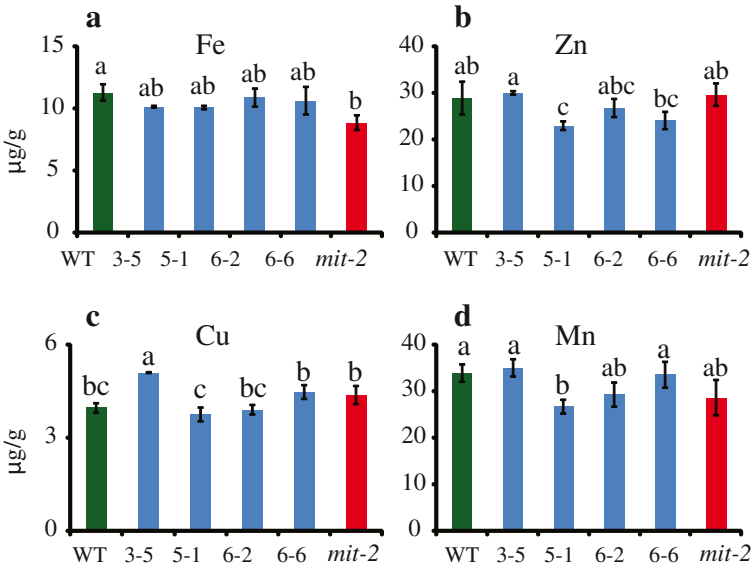

## White rice

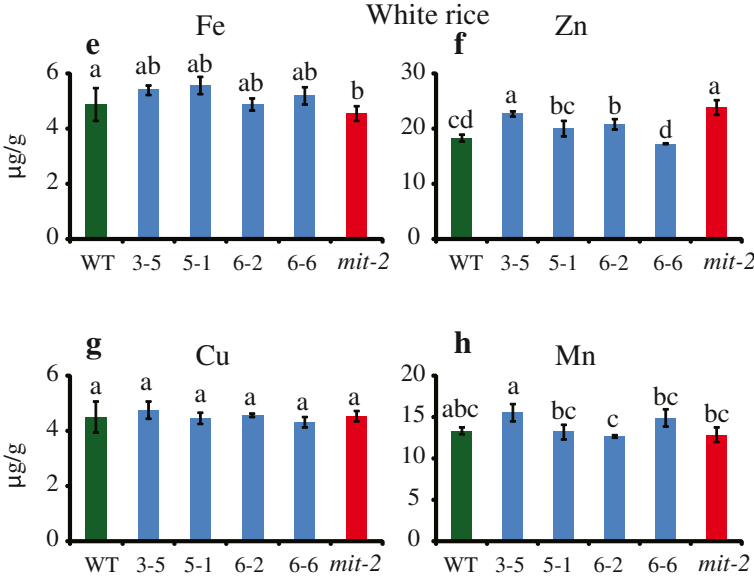

Supplement: Supplementary file 7 — Authors’ original file for figure 6 [file 12284_2013_65_MOESM7_ESM.pdf]
